# Tumor Size Modifies the Survival Benefit of Chemotherapy in Localized Soft Tissue Sarcomas: A Propensity-Matched Cohort Study

**DOI:** 10.3390/jcm15062253

**Published:** 2026-03-16

**Authors:** Kole Joachim, Brandon Gettleman, Michael Fice, Adrian Lin, Christopher David Hamad, Othneil Sparks, Ezekiel Dingle, Casey Abernethy, Nicholas M. Bernthal, Alexander B. Christ

**Affiliations:** 1 David Geffen School of Medicine, University of California, Los Angeles, CA 90095, USA; kjoachim@mednet.ucla.edu (K.J.); adrianlin@mednet.ucla.edu (A.L.); osparks@mednet.ucla.edu (O.S.); edingle@mednet.ucla.edu (E.D.); cabernethy@mednet.ucla.edu (C.A.); 2 Department of Orthopaedic Surgery, University of California, Los Angeles, CA 90095, USA; bgettleman@mednet.ucla.edu (B.G.); mfice13@gmail.com (M.F.); nbernthal@mednet.ucla.edu (N.M.B.); 3 Department of Orthopaedic Surgery, Greater Los Angeles VA Medical Center, Los Angeles, CA 90073, USA

**Keywords:** undifferentiated pleomorphic sarcoma, dedifferentiated liposarcoma, fibromyxosarcoma, epithelioid sarcoma, synovial sarcoma, chemotherapy

## Abstract

**Background/Introduction:** Soft tissue sarcomas (STS) represent a diverse group of rare cancers that have variable responses to chemotherapy. Although tumor size is an established prognostic factor, its influence on the benefit of chemotherapy within specific histologies is not well understood. **Methods:** We conducted a retrospective analysis of 3890 patients with five STS subtypes using SEER data from 2000 to 2021. Patients were stratified by tumor size (<5 cm, 5–10 cm, >10 cm) and propensity score matched within each subtype-size cohort to control for confounders. Cox regression assessed the impact of chemotherapy on overall survival, with results presented as hazard ratios (HR) and 95% confidence intervals (95%-CI). Inverse probability of treatment weighting (IPTW) was used to improve selection bias. **Results:** Chemotherapy use in UPS demonstrated worse survival in smaller tumors <5 cm (HR = 2.65, 95%-CI = 1.19–5.92, *p* = 0.018) and 5–10 cm tumors (HR = 1.45, 95%-CI = 1.03–2.04, *p* = 0.031). In larger UPS tumors (>10 cm), a directionally protective association observed in matched analysis attenuated after inverse probability of treatment weighting (IPTW) (HR = 0.82, 95%-CI = 0.60–1.12, *p* = 0.211). Fibromyxosarcoma 5–10 cm tumors demonstrated worse survival with chemotherapy (matched HR = 3.74, 95%-CI = 2.30–6.10, *p* < 0.001), which remained consistent after IPTW (HR = 4.47, 95%-CI = 2.63–7.60, *p* < 0.001), along with >10 cm tumors (IPTW HR = 2.16, 95%-CI = 1.07–4.34, *p* = 0.031). DDLPS >10 cm tumors demonstrated a directionally harmful association (HR = 1.49, 95%-CI = 0.96–2.29, *p* = 0.073). Synovial sarcoma 5–10 cm tumors demonstrated a directionally protective trend that remained statistically non-significant across analyses. **Conclusions:** The effect of chemotherapy on survival in localized STS depends on both histologic subtype and tumor size. However, subgroup estimates with confidence intervals approaching 1.0 should be interpreted cautiously.

## 1. Introduction

Soft tissue sarcomas (STS) are a rare and diverse group of cancers that develop from mesenchymal tissues. They include over 50 different histological subtypes, each with unique biological behaviors and responses to treatment [1,2,3,4]. Among these, subtypes such as undifferentiated pleomorphic sarcoma (UPS), dedifferentiated liposarcoma (DDLPS), fibromyxosarcoma, epithelioid sarcoma, and synovial sarcoma have distinct histologic and molecular features that complicate pooled analyses in sarcoma research. While surgical wide excision and radiotherapy remain the primary treatment modalities for localized disease, chemotherapy is inconsistently utilized in the setting of local recurrence or disease deep to the investing fascia. However, growing evidence demonstrates that chemotherapy effectiveness varies among sarcoma subtypes, with some being highly sensitive to treatment and others demonstrating minimal response to cytotoxic drugs [5,6,7].

This recognition of subtype-specific treatment responses has led to landmark studies such as the ISG-STS 1001 trial, which compared histology-tailored chemotherapy regimens to standard anthracycline-ifosfamide combinations in five major sarcoma subtypes, highlighting the complexity of personalized treatment approaches [8,9]. The complexity emphasized by this trial reflects broader patterns in sarcoma treatment, where published chemotherapy response rates vary from 10 to 50% depending on patient selection, tumor grade, and histological subtype [10]. Despite this increasing awareness of histologic heterogeneity, current treatment algorithms do not account for how tumor characteristics like size may further stratify chemotherapy benefits within individual subtypes. While tumor size remains a well-established prognostic factor, its role as a modifier of therapeutic response within specific histologies is still poorly understood [11]. This important knowledge gap limits our ability to offer truly personalized treatment recommendations and underscores the need for subtype- and size-based analyses in various sarcoma populations.

In this study, we aimed to evaluate the impact of chemotherapy on overall survival across five rare soft tissue sarcoma subtypes, stratified by tumor size. Using propensity score matching and Inverse probability of treatment weighting (IPTW) to reduce confounding and treatment selection bias, we created 15 distinct subtype-size cohorts to isolate treatment effects. Our primary objective was to determine whether chemotherapy provided a survival benefit within each histologic subtype. A secondary goal was to assess whether tumor size influenced this treatment effect, offering a more detailed understanding of chemotherapy responsiveness across clinically relevant patient subgroups.

## 2. Materials and Methods

### 2.1. Study Design and Data Source

We conducted a retrospective cohort analysis of patients diagnosed with rare soft tissue sarcomas, including undifferentiated pleomorphic sarcoma, dedifferentiated liposarcoma, fibromyxosarcoma, epithelioid sarcoma, and synovial sarcoma, between 2000 and 2021. Data were obtained from the Surveillance, Epidemiology, and End Results (SEER) database, a population-based collection of individual-level data. Patients from the November 2023 SEER dataset submission were queried using SEER*Stat 8.4.4. This submission included patients from 17 registries diagnosed with cancer from 2000 to 2021 and represents roughly 26.5% of the US population [12].

### 2.2. Patient Selection

A total of 3890 patients with five soft tissue sarcoma subtypes were included: undifferentiated pleomorphic sarcoma (*n* = 1627, 41.8%), dedifferentiated liposarcoma (*n* = 615, 15.8%), fibromyxosarcoma (*n* = 1255, 32.3%), epithelioid sarcoma (*n* = 156, 4.0%), and synovial sarcoma (*n* = 237, 6.1%). Only cases with histologically confirmed diagnoses were included in the analysis. Patients were excluded if they had metastatic disease at presentation.

### 2.3. Variable Definitions

Patient demographics included: age grouped into four categories (young [0–49 years], middle-aged [50–69 years], older [70–79 years], and elderly [≥80 years]), sex, and tumor characteristics such as histologic grade (1–4) and tumor size. Tumor size was divided into three categories: small (<5 cm), medium (5–10 cm), and large (>10 cm). Treatment variables included receipt of chemotherapy, surgical resection, and radiotherapy utilization. Chemotherapy exposure was defined using the SEER chemotherapy recode variable, which categorizes treatment as “yes” versus “no/unknown.” Because SEER does not distinguish between patients who definitively did not receive chemotherapy and those with missing treatment documentation, exclusion of unknown cases was not possible. Accordingly, chemotherapy was analyzed as documented receipt versus no/unknown, and results were interpreted as associations subject to potential treatment misclassification. The primary outcome was overall survival, measured in months from diagnosis to death.

### 2.4. Statistical Analysis

To address selection bias in chemotherapy administration, propensity score matching was conducted using a probit regression model. Propensity scores were derived from factors such as age group, sex, tumor grade, surgical status, and radiotherapy utilization. One-to-one nearest-neighbor matching without replacement was performed using a caliper width of 0.2 on the propensity score scale. Analyses were performed separately within each sarcoma subtype-tumor size cohort. Covariate balance after matching was evaluated using standardized mean differences for all matching variables, along with summary balance metrics including Rubin’s B statistic. Matching success was assessed by reporting the number of patients retained within each matched cohort.

Analyses were stratified by both sarcoma subtype and tumor size category, resulting in 15 distinct cohorts. For each matched cohort, baseline characteristics and standardized differences were examined to assess balance between chemotherapy and non-chemotherapy groups. Patients lacking common support were excluded from survival analyses.

Survival analysis was conducted using Cox proportional hazards regression on the matched cohorts. Hazard ratios (HR) and 95% confidence intervals (95% CI) were calculated to assess the impact of chemotherapy on overall survival within each sarcoma subtype-tumor size cohort. Kaplan–Meier survival curves were generated for selected cohorts to visualize treatment effects. As a sensitivity analysis, inverse probability of treatment weighting (IPTW) was performed to evaluate treatment effects using the entire cohort rather than matched samples. Propensity scores representing the probability of receiving chemotherapy were estimated separately within each histology and tumor size cohort using multivariable logistic regression including age group, sex, tumor grade, surgery to the primary site, and receipt of radiation therapy. In cohorts with sparse data leading to model instability or separation, tumor grade was collapsed into a binary high-grade versus low-grade variable. Stabilized IPTW weights were calculated using the marginal probability of treatment and truncated at the 1st and 99th percentiles to limit the influence of extreme weights. Weighted Cox proportional hazards models with standard errors were then used to estimate the association between chemotherapy and overall survival. Given the absence of certain clinical covariates within SEER, IPTW estimates were interpreted as adjusted associations rather than causal effects. All statistical analyses were performed using Stata 18 SE (StataCorp LLC, College Station, TX, USA). Given the prespecified stratification into 15 histology-tumor size cohorts, we emphasize effect sizes (HRs) and 95% confidence intervals rather than dichotomous significance thresholds. *p*-values are reported to describe the strength of evidence but should be interpreted cautiously in the setting of multiple subgroup comparisons.

## 3. Results

### 3.1. Undifferentiated Pleomorphic Sarcoma

**Figure 1:**
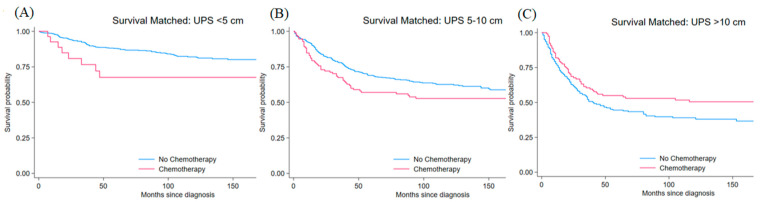
Kaplan–Meier survival curves for matched undifferentiated pleomorphic sarcoma patients comparing chemotherapy (red line) versus no chemotherapy (blue line) (**A**) <5 cm tumors (HR 2.07, 95% CI 1.00–4.28, *p* = 0.050), (**B**) 5–10 cm tumors (HR 1.37, 95% CI 1.00–1.87, *p* = 0.049), and (**C**) >10 cm tumors (HR 0.72, 95% CI 0.53–0.99, *p* = 0.041). Time is measured in months from diagnosis. Estimates with confidence intervals approaching 1.0 should be interpreted cautiously.

UPS comprised the largest group, with 1627 patients (41.8% of the total). Most patients were elderly (≥80 years, 59.3%) or older adults (70–79 years, 35.5%), with a male predominance (58.9% vs. 41.1%, *p* < 0.001). The majority underwent surgical resection (92.9% vs. 7.1%, *p* < 0.001), while only a small percentage received chemotherapy (17.5% vs. 82.5%, *p* < 0.001). Radiotherapy was used in 61.6% of patients. Tumor size was relatively evenly distributed: 34.3% (<5 cm), 38.8% (5–10 cm), and 26.9% (>10 cm). High-grade tumors were most common, with 55.5% grade 4 and 31.0% grade 3 lesions (Table 1). After propensity score matching, 549 patients with small tumors, 625 with medium tumors, and 408 with large tumors were included in the survival analysis. Post-matching standardized differences for tumors <5 cm were 4.6% for age, 13.9% for sex, 4.5% for grade, 11.1% for surgery status, and 0.0% for radiotherapy. For tumors 5–10 cm, standardized differences were 0.0% for age, 1.8% for sex, 1.5% for grade, 3.2% for surgery status, and 1.9% for radiotherapy. For tumors >10 cm, standardized differences were 4.4% for age, 8.2% for sex, 0.0% for grade, 7.5% for surgery status, and 1.7% for radiotherapy. Propensity score matching improved baseline covariate balance (mean bias reduced from 37.0% to 6.8% for <5 cm, 25.0% to 1.7% for 5–10 cm, and 27.1% to 4.4% for >10 cm). Global balance metrics also improved after matching (Rubin’s B decreased from 97.2 to 18.4 for <5 cm; 70.0 to 4.6 for 5–10 cm; and 88.2 to 14.1 for >10 cm). In matched UPS cohorts, chemotherapy showed borderline harmful associations in <5 cm tumors (HR 2.07, 95% CI 1.00–4.28; *p* = 0.050) and 5–10 cm tumors (HR 1.37, 95% CI 1.00–1.87; *p* = 0.049), with confidence intervals that approached 1.0. For >10 cm tumors, chemotherapy was associated with a directionally protective estimate in matched analysis (HR 0.72, 95% CI 0.53–0.99; *p* = 0.041). Kaplan–Meier survival curves stratified by tumor size are shown in Figure 1. Inverse probability of treatment weighting yielded hazard ratios of 2.65 (95% CI: 1.19–5.92, *p* = 0.018) for tumors < 5 cm, 1.45 (95% CI: 1.03–2.04, *p* = 0.031) for tumors 5–10 cm, and 0.82 (95% CI: 0.60–1.12, *p* = 0.211) for tumors > 10 cm (Table 2).

### 3.2. Dedifferentiated Liposarcoma

**Figure 2:**
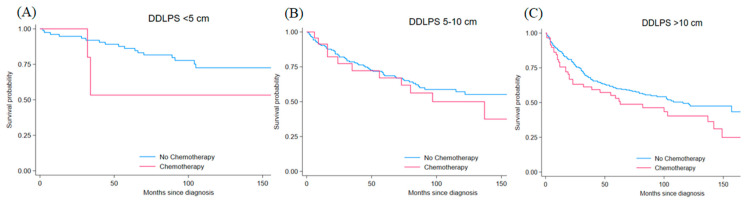
Kaplan–Meier Survival Curves for Matched Dedifferentiated Liposarcoma (DDLPS) comparing chemotherapy (red line) versus no chemotherapy (blue line) (**A**) <5 cm tumors (HR 2.17, 95% CI 0.50–9.47, *p* = 0.303), (**B**) 5–10 cm tumors (HR 1.24, 95% CI 0.65–2.37, *p* = 0.508), and (**C**) >10 cm tumors (HR 1.42, 95% CI 0.97–2.08, *p* = 0.069). Time is measured in months from diagnosis. Estimates with confidence intervals approaching 1.0 should be interpreted cautiously.

DDLPS involved 615 patients (15.8% of the total), with 55.5% being elderly and 40.7% older adults, showing a large male predominance (68.6% vs. 31.4%, *p* < 0.001). Most patients underwent surgery (94.8% vs. 5.2%, *p* < 0.001), while 15.3% received chemotherapy and 50.2% received radiotherapy. Tumors larger than 10 cm were most common (56.1% vs. 13.8% for <5 cm and 30.1% for 5–10 cm, *p* < 0.001). The majority had high-grade lesions, with 43.4% grade 4, 37.4% grade 3, 11.9% grade 2, and 7.3% grade 1 (*p* < 0.001) (Table 1). Matched cohorts included 84 patients with <5 cm tumors, 180 with 5–10 cm tumors, and 337 with >10 cm tumors. For tumors < 5 cm, post-matching standardized differences were 0.0% for age, sex, grade, and radiotherapy, with surgery status identical between groups. For tumors 5–10 cm, standardized differences after matching were 7.6% for age, 0.0% for sex, 20.3% for grade, 30.4% for surgery status, and 35.0% for radiotherapy. For tumors > 10 cm, standardized differences were 8.9% for age, 0.0% for sex, 5.7% for grade, 19.2% for surgery status, and 20.3% for radiotherapy. Matching improved balance across size cohorts, although performance varied by tumor size. For <5 cm tumors, matching produced exact covariate balance on measured variables but retained only 6 chemotherapy-treated patients. For 5–10 cm tumors, balance improved (mean bias 43.9% to 18.6%, Rubin’s B: 127.2 to 47.9), whereas for >10 cm tumors balance improved more consistently (mean bias 21.0% to 10.8%; Rubin’s B: 64.0 to 27.0). Chemotherapy was not associated with a significant survival difference for small tumors (HR = 2.17, 95% CI: 0.50–9.47, *p* = 0.303) or medium tumors (HR = 1.24, 95% CI: 0.65–2.37, *p* = 0.508). However, in large tumors, chemotherapy trended toward worse survival (HR = 1.42, 95% CI: 0.97–2.08, *p* = 0.069). Kaplan–Meier survival curves stratified by tumor size are shown in Figure 2. After IPTW, chemotherapy remained unassociated with survival across tumor sizes, including <5 cm (HR 1.90, 95% CI 0.40–9.04, *p* = 0.418), 5–10 cm (HR 0.78, 95% CI 0.36–1.66, *p* = 0.514), and >10 cm tumors (HR 1.49, 95% CI 0.96–2.29, *p* = 0.073) (Table 2).

### 3.3. Fibromyxosarcoma

**Figure 3:**
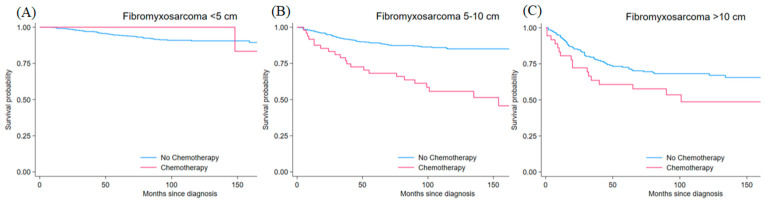
Kaplan–Meier survival curves for matched fibromyxosarcoma patients by tumor size comparing chemotherapy (red line) versus no chemotherapy (blue line) (**A**) <5 cm tumors (HR 0.85, 95% CI 0.12–6.16, *p* = 0.870), (**B**) 5–10 cm tumors (HR 3.74, 95% CI 2.30–6.10, *p* < 0.001), and (**C**) >10 cm tumors (HR 1.72, 95% CI 1.01–2.96, *p* = 0.048). Time is measured in months from diagnosis. Estimates with confidence intervals approaching 1.0 should be interpreted cautiously.

Fibromyxosarcoma involved 1255 patients, accounting for 32.3% of the total. The age distribution showed 45.4% elderly, 39.8% older adults, 10.9% middle-aged, and 3.8% young patients. The sex distribution indicated a modest male predominance (53.1% vs. 46.9%, *p* < 0.001). Nearly all patients underwent surgery (97.2% vs. 2.8%, *p* < 0.001), while only 8.1% received chemotherapy and 53.6% received radiotherapy. Tumor sizes were relatively evenly distributed between <5 cm (40.3%) and 5–10 cm (40.5%), with fewer tumors larger than 10 cm (19.2%, *p* < 0.001). Grade distribution was more varied, with 15.5% grade 1, 37.7% grade 2, 16.9% grade 3, and 30.0% grade 4 (*p* < 0.001) (Table 1). Matched analyses included 499 patients with tumors <5 cm, 505 with tumors 5–10 cm, and 235 with tumors > 10 cm. For tumors < 5 cm, post-matching standardized differences were 20.0% for age, 16.3% for sex, 7.6% for grade, identical surgical status between groups, and 0.0% for radiotherapy. For tumors 5–10 cm, standardized differences after matching were 2.7% for age, 4.2% for sex, 4.4% for grade, 9.6% for surgery status, and 4.4% for radiotherapy. For tumors > 10 cm, standardized differences were 8.6% for age, 0.0% for sex, 16.8% for grade, 0.0% for surgery status, and 19.7% for radiotherapy. Propensity score matching improved baseline balance, particularly for 5–10 cm and >10 cm tumors (mean bias reduced from 40.0% to 5.0% for 5–10 cm [Rubin’s B: 118.2 to 13.2] and from 40.6% to 9.0% for >10 cm [Rubin’s B: 119.6 to 36.7]). For <5 cm tumors, matching reduced mean bias from 41.1% to 11.0% (Rubin’s B: 95.4 to 31.3). For small tumors, chemotherapy was not associated with survival differences (HR = 0.85, 95% CI: 0.12–6.16, *p* = 0.870). However, chemotherapy was associated with worse survival in 5–10 cm tumors (HR 3.74, 95% CI 2.30–6.10; *p* < 0.001). In >10 cm tumors, the estimate was directionally harmful with borderline evidence in matched analysis (HR 1.72, 95% CI 1.01–2.96; *p* = 0.048). Kaplan–Meier survival curves stratified by tumor size are shown in Figure 3. After IPTW, hazard ratios were 0.58 (95% CI 0.08–4.02, *p* = 0.579) for tumors < 5 cm, 4.47 (95% CI 2.63–7.60, *p* < 0.001) for tumors 5–10 cm, and 2.16 (95% CI 1.07–4.34, *p* = 0.031) for tumors >10 cm (Table 2).

### 3.4. Epithelioid Sarcoma

**Figure 4:**
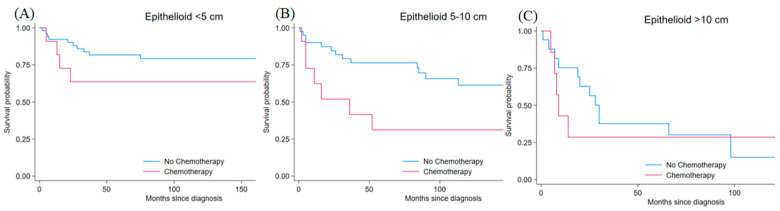
Kaplan–Meier survival curves for matched epithelioid sarcoma patients by tumor size comparing chemotherapy (red line) versus no chemotherapy (blue line) (**A**) <5 cm tumors (HR 2.07, 95% CI 0.65–6.60, *p* = 0.220), (**B**) 5–10 cm tumors (HR 2.50, 95% CI 1.00–6.24, *p* = 0.050), and (**C**) >10 cm tumors (HR 1.30, 95% CI 0.45–3.72, *p* = 0.631). Time is measured in months from diagnosis. Estimates with confidence intervals approaching 1.0 should be interpreted cautiously.

Epithelioid sarcoma represented the smallest cohort with 156 patients (4.0% of the total), exhibiting a wide age range: 40.4% were older adults, 25.6% elderly, 25.0% middle-aged, and 9.0% young patients. Males accounted for the majority of cases (57.7% vs. 42.3%, *p* < 0.001). Surgery rates were lower (84.0% vs. 16.0%, *p* < 0.001) compared to other subtypes, while chemotherapy use was higher (28.2% vs. 71.8%, *p* < 0.001). Radiotherapy was administered in 57.1% of patients. Tumor size distribution showed 41.7% <5 cm, 36.5% 5–10 cm, and 21.8% >10 cm tumors. High-grade tumors were predominant, with 48.7% grade 4, 35.9% grade 3, 12.2% grade 2, and 3.2% grade 1 (*p* < 0.001) (Table 1). The matched analysis included 63 patients with tumors < 5 cm, 52 with tumors 5–10 cm, and 24 with tumors > 10 cm. For tumors < 5 cm, post-matching standardized differences were 9.4% for age, 0.0% for sex, 20.4% for grade, 0.0% for surgery status, and 35.5% for radiotherapy. For tumors 5–10 cm, standardized differences were 11.2% for age, 18.0% for sex, 27.8% for grade, 0.0% for surgery status, and 0.0% for radiotherapy. For tumors > 10 cm, standardized differences were 16.2% for age, 30.9% for sex, 20.2% for grade, 33.3% for surgery status, and 0.0% for radiotherapy. For <5 cm tumors, matching reduced mean bias from 22.1% to 13.1% with Rubin’s B decreasing from 87.2 to 46.5. For 5–10 cm tumors, mean bias improved from 35.7% to 11.4% and Rubin’s B decreased from 111.5 to 46.6, while for >10 cm tumors mean bias improved from 43.3% to 20.1% but Rubin’s B remained high (132.5 to 104.6). Chemotherapy was not significantly associated with survival differences for small tumors (HR = 2.07, 95% CI: 0.65–6.60, *p* = 0.220) or large tumors (HR = 1.30, 95% CI: 0.45–3.72, *p* = 0.631), while the 5–10 cm cohort showed a borderline harmful association in matched analysis (HR 2.50, 95% CI 1.00–6.24; *p* = 0.050). Kaplan–Meier survival curves stratified by tumor size are shown in Figure 4. After IPTW, hazard ratios were 1.97 (95% CI 0.58–6.70, *p* = 0.277) for tumors < 5 cm, 2.38 (95% CI 0.92–6.18, *p* = 0.075) for tumors 5–10 cm, and 0.89 (95% CI 0.38–2.06, *p* = 0.782) for tumors >10 cm (Table 2).

### 3.5. Synovial Sarcoma

**Figure 5:**
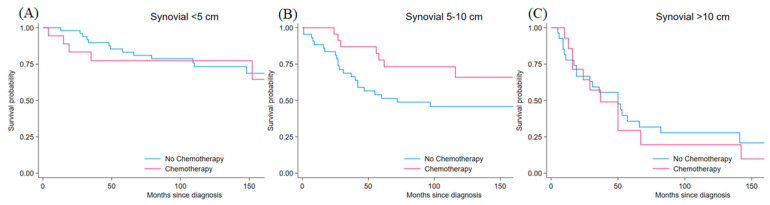
Kaplan–Meier survival curves for matched synovial sarcoma patients by tumor size comparing chemotherapy (red line) versus no chemotherapy (blue line) (**A**) <5 cm tumors (HR 1.13, 95% CI 0.40–3.18, *p* = 0.813), (**B**) 5–10 cm tumors (HR 0.45, 95% CI 0.19–1.05, *p* = 0.063), and (**C**) >10 cm tumors (HR 1.20, 95% CI 0.57–2.52, *p* = 0.631). Time is measured in months from diagnosis. Estimates with confidence intervals approaching 1.0 should be interpreted cautiously.

Synovial sarcoma comprised 237 patients (6.1% of the total) and demonstrated the youngest age distribution among all subtypes: 35.9% were older adults, 35.9% middle-aged, 15.2% young, and 13.1% elderly. The sex distribution revealed a slight male predominance (54.9% vs. 45.1%, *p* < 0.001). Surgery was performed in 86.1% (vs 13.9% no surgery), and chemotherapy use was 47.7%. Radiotherapy was utilized in 63.3% of patients. Tumor size distribution indicated 29.1% had tumors < 5 cm, 42.6% had 5–10 cm tumors, and 28.3% had tumors > 10 cm (*p* < 0.001). High-grade lesions predominated, with 49.4% grade 3, 27.4% grade 4, 20.7% grade 2, and 2.5% grade 1 (*p* < 0.001) (Table 1). Matched cohorts included 69 patients with tumors < 5 cm, 68 with tumors 5–10 cm, and 41 with tumors > 10 cm. For tumors < 5 cm, post-matching standardized differences were 11.6% for age, 10.9% for sex, 37.0% for grade, 28.7% for surgery status, and 11.5% for radiotherapy. For tumors 5–10 cm, standardized differences were 35.3% for age, 8.3% for sex, 26.3% for grade, 12.0% for surgery status, and 8.9% for radiotherapy. For tumors > 10 cm, standardized differences were 17.7% for age, 0.0% for sex, 10.2% for grade, 20.8% for surgery status, and 0.0% for radiotherapy. Mean bias decreased from 24.6% to 19.9% for <5 cm tumors, from 40.5% to 18.1% for 5–10 cm tumors, and from 45.0% to 9.7% for >10 cm tumors. Rubin’s B decreased from 82.0 to 49.0 for <5 cm tumors, from 119.6 to 47.6 for 5–10 cm tumors, and from 150.2 to 58.0 for >10 cm tumors. For tumors <5 cm, chemotherapy showed no survival difference (HR = 1.13, 95% CI: 0.40–3.18, *p* = 0.813). For tumors 5–10 cm, chemotherapy showed a trend toward improved survival (HR = 0.45, 95% CI: 0.19–1.05, *p* = 0.063). For tumors >10 cm, no significant difference was observed (HR = 1.20, 95% CI: 0.57–2.52, *p* = 0.631). Kaplan–Meier survival curves stratified by tumor size are shown in Figure 5. After IPTW, hazard ratios were 0.69 (95% CI 0.21–2.27, *p* = 0.543) for tumors < 5 cm, 0.56 (95% CI 0.22–1.44, *p* = 0.232) for tumors 5–10 cm, and 1.38 (95% CI 0.78–2.42, *p* = 0.268) for tumors > 10 cm (Table 2).

## 4. Discussion

This study presents a large, propensity-matched analysis of five sarcoma subtypes and their differential response to chemotherapy stratified by tumor size. Although tumor size is a well-established prognostic factor in soft tissue sarcoma, its role as a modifier of chemotherapy effectiveness is not well understood, especially within individual histologic subtypes. To address this gap, we tested whether chemotherapy response varied across tumor size categories within each non-metastatic sarcoma subtype using propensity score matching to balance for age, sex, tumor grade, surgical status, and radiation treatment. We created size- and subtype-specific matched groups to reduce confounding by indication and to better isolate the treatment effect of chemotherapy. These subgroups were checked via Rubin’s B values to determine the cohort matching quality, followed by an inverse probability weighted analysis to improve the clarity of the results. This method enabled us to isolate the effects of tumor size and chemotherapy efficacy on initial prognosis varied by sarcoma subtype, providing evidence in support of histology-specific chemotherapy treatment choices for sarcomas.

Across subtype and size-stratified analyses, chemotherapy associations varied by tumor size, with UPS and fibromyxosarcoma demonstrating the most consistent size-dependent patterns after IPTW adjustment. In IPTW analyses, the UPS > 10 cm association remained directionally protective but no longer met statistical significance. This attenuation suggests that apparent benefit in large UPS tumors may not be uniform across all patients but instead reflects treatment-selection factors not captured within registry data. In clinical practice, patients selected for chemotherapy may differ systematically from untreated patients with respect to functional status, tumor behavior, or other unmeasured clinical characteristics. Because these factors are incompletely captured in SEER, residual confounding may influence observed survival patterns despite propensity matching and weighting. The attenuation observed after IPTW therefore supports an interpretation in which treatment selection enriches for patients more likely to experience favorable outcomes rather than chemotherapy conferring equivalent benefit across all large UPS cases. The divergent chemotherapy associations observed among sarcoma subtypes and tumor sizes may also reflect underlying biological heterogeneity. Although our registry-based analysis cannot directly evaluate molecular mechanisms, differences in tumor proliferation, genomic instability, and treatment sensitivity have been proposed as potential contributors to variable chemotherapy responsiveness across sarcoma subtypes. For UPS tumors >10 cm, the directionally protective trend compared with the observed harm in smaller tumors raises the possibility that larger lesions may represent biologically distinct subsets, such as tumors with higher proliferative activity or clonal evolution favoring chemotherapy susceptibility [13,14]. Prior molecular studies have suggested that subsets of UPS demonstrate activation of cell-cycle and stemness pathways with relative immune exclusion, features that may enhance sensitivity to DNA-damaging agents such as anthracyclines and platinum compounds [15,16,17,18,19]. These biological hypotheses should therefore be viewed as contextual interpretations rather than conclusions derived from the present dataset. Synovial sarcoma demonstrated a directionally protective association with chemotherapy in medium-sized tumors, although this finding did not reach statistical significance and attenuated after weighting analyses. The observed trend is nevertheless biologically plausible given the unique molecular characteristics of synovial sarcoma. Unlike several other soft tissue sarcoma subtypes, synovial sarcoma retains relatively intact p53-mediated apoptotic signaling and is driven by the SS18-SSX fusion protein, which induces widespread epigenetic remodeling that may enhance susceptibility to cytotoxic agents [16,17,18,19,20]. In addition, synovial sarcoma frequently expresses the cancer-testis antigen NY-ESO-1, reported in approximately 70–80% of cases, reflecting a comparatively immunogenic tumor microenvironment. This feature has enabled development of NY-ESO-1-targeted cellular therapies that have demonstrated encouraging clinical activity and highlight the broader therapeutic vulnerability of this subtype beyond conventional chemotherapy [19,21,22,23,24,25,26]. Taken together, these biological features may help explain why chemotherapy responsiveness in synovial sarcoma has been observed in prior studies, while the uncertainty seen in our analysis underscores the need for prospective validation and molecularly stratified treatment selection.

Conversely, the subtypes with poor chemotherapy responses possess distinct resistance mechanisms. DDLPS exhibits MDM2 amplification, directly targeting p53 for degradation, which is thought to contribute to the bypass of chemotherapy-induced apoptosis [27,28]. This is supported by the lack of statistically significant associations across sizes, before and after treatment weighting. Epithelioid sarcoma demonstrated a lack of consistent association between chemotherapy and survival across tumor size groups, with estimates characterized by wide confidence intervals and attenuation after inverse probability weighting. In matched analyses, medium-sized tumors showed a borderline harmful association that diminished after weighting, suggesting sensitivity of the estimate to treatment-selection factors. Biologically, epithelioid sarcoma is characterized by loss of SMARCB1, which disrupts chromatin remodeling complexes and has been associated with relative resistance to conventional cytotoxic chemotherapy [29,30]. The absence of a stable treatment signal in our analysis is therefore consistent with known resistance mechanisms, although small cohort sizes and low event counts limit definitive conclusions. Taken together, these findings highlight the need for prospective or molecularly stratified studies in this rare subtype. Fibromyxosarcoma’s myxoid features and low proliferative rates make cytotoxic agents less effective against slowly dividing cells [31,32]. Interestingly, fibromyxosarcoma demonstrated a different pattern in sensitivity analyses compared with UPS. Inverse probability weighting produced estimates that were consistent in direction and remained statistically significant in the intermediate- and large-tumor cohorts, indicating that the observed association was not substantially altered after adjustment for measured treatment-selection factors. This stability across analytic approaches suggests that poorer outcomes among treated patients are less likely to reflect treatment-selection bias and more likely to represent intrinsic chemotherapy resistance within this subtype. Given the relatively low proliferative activity and abundant myxoid stroma characteristic of fibromyxosarcoma, these findings are consistent with reduced sensitivity to cytotoxic agents.

Our findings extend previous registry-based analyses that suggest chemotherapy in UPS mainly benefits patients with large, high-risk tumors. For example, a Japanese population-based cohort of 2112 patients with localized UPS showed that adjuvant chemotherapy did not improve survival for tumors < 5 cm, had a marginal effect on tumors 5–10 cm, and was most effective in the 10–15 cm range after propensity score matching [33]. This aligns with our observation of a directionally protective association for UPS tumors >10 cm (HR 0.72), despite the loss of significance after IPTW. Taken together, these findings suggest that tumor size in UPS may function less as a direct predictor of chemotherapy response and more as a clinical surrogate for underlying tumor biology. Larger tumors that progress despite prolonged growth may represent genomically unstable but chemotherapy-susceptible clones, whereas smaller UPS lesions may include biologically indolent or therapy-resistant tumors for which systemic therapy provides limited survival advantage. The differential findings between matched and IPTW analyses are therefore consistent with biologic enrichment rather than statistical artifact. A review by the European Organisation for Research and Treatment of Cancer (EORTC) reported response rates of 27.8% in synovial sarcoma compared to 18.8% in other soft tissue sarcomas [34]. Notably, retrospective analyses show that medium-sized synovial sarcomas (5–10 cm) derive significant benefits from ifosfamide-based chemotherapy, with improved disease-specific survival and distant recurrence-free survival, especially in tumors within this size range [35,36]. In our data, the propensity matched analysis showed a trend toward improved survival for 5–10 cm synovial sarcomas (HR 0.45, 95% CI 0.19–1.05, *p* = 0.063), but this association did not persist after treatment weighting (HR 0.56, 95% CI 0.22–1.44, *p* = 0.232). Thus, while our findings are directionally consistent with prior reports, they are tempered by loss of significance after adjustment for measured treatment selection factors, underscoring the need for prospective validation and molecular or response biomarkers to identify the patients most likely to benefit.

There are several strengths of this study worth highlighting. It represents one of the most comprehensive real-world evaluations of chemotherapy effectiveness across five soft tissue sarcoma subtypes. A key methodological strength is the use of propensity score matching across 15 distinct cohorts defined by both histologic subtype and tumor size. This approach allows for rigorous adjustment for confounding factors and treatment selection bias, while helping estimate treatment effects within clinically relevant cohorts. Unlike previous analyses that combine heterogeneous sarcoma populations, our stratified design uncovers subtype- and size-specific patterns of therapeutic benefit, neutrality, or harm that might otherwise remain hidden. These findings offer a more precise evidence base to guide personalized chemotherapy decisions in managing rare sarcomas. However, several statistical considerations should be acknowledged when interpreting these findings. This study evaluated chemotherapy associations across 15 prespecified histology-tumor size cohorts, and multiple subgroup comparisons increase the likelihood of borderline *p*-values occurring by chance. Accordingly, results with *p*-values near 0.05 and confidence intervals approaching 1.0 should be interpreted cautiously and not as definitive threshold-based positives. We therefore emphasize the magnitude, direction, and precision of hazard ratios, as well as consistency across analytic approaches, rather than reliance on dichotomous significance thresholds. Accordingly, subgroup findings that attenuated after weighting are best interpreted as hypothesis-generating. Several histology-tumor size cohorts were small, particularly rare subtypes such as epithelioid sarcoma and synovial sarcoma, resulting in limited event counts and wide confidence intervals. Estimates from these cohorts should therefore be interpreted as exploratory rather than definitive effect estimates. Even after propensity score matching to reduce selection bias, some confounding might remain if patients with large, symptomatic tumors receiving chemotherapy had unmeasured traits linked to better prognosis, like better performance status or more aggressive tumor biology that is paradoxically more responsive to chemotherapy. The SEER database lacks important clinical details such as performance status, treatment intent, and chemotherapy regimen, which limits our ability to evaluate dosing strategies or compare neoadjuvant versus adjuvant effects. Accordingly, observed associations should be interpreted as reflecting chemotherapy exposure in general rather than regimen-specific treatment effects, which may vary across sarcoma subtypes and institutions. Chemotherapy in these populations is not standardized across centers or treating physicians, and therefore we expect some unmeasurable variability between patients that could affect outcomes. Additionally, histologic classification within SEER reflects registry-based ICD-O coding rather than centralized pathology review. For example, fibromyxosarcoma (ICD-O-3 code 8811/3) may include biologically related tumors that would be separated under contemporary classification systems, introducing some degree of histologic heterogeneity [37,38]. Because this type of variation is unlikely to be systematically related to whether patients received chemotherapy, it would generally make true treatment differences harder to detect rather than create consistent associations on its own. In our analysis, chemotherapy associations within fibromyxosarcoma remained present after inverse probability of treatment weighting and continued to reach significance in the intermediate- and large-tumor cohorts, suggesting that differences in treatment selection rather than registry coding alone are more likely to explain the observed findings. Also, overall survival was the only outcome measured, without including quality-of-life or progression metrics. Given sarcoma populations in SEER are predominantly older adults, age-related differences in physiologic reserve and treatment tolerance may influence chemotherapy selection and outcomes. Although age was incorporated into propensity score matching and IPTW models to balance treated and untreated groups, residual confounding related to functional status or frailty may persist. Taken together, the differing behavior of UPS and fibromyxosarcoma across analytic approaches suggests that apparent chemotherapy effects in registry data may reflect two distinct realities: biologically enriched treatment benefit in certain subtypes and intrinsic therapeutic resistance in others. These limitations highlight the need for prospective, biomarker-driven validation in future research. While the biological basis has yet to be elucidated, the consistent size-dependent patterns across subtypes highlight the need for more research into how tumor size and burden influence chemotherapy effectiveness in soft tissue sarcomas.

## 5. Conclusions

In this population-based SEER cohort, the association between chemotherapy and survival in localized soft tissue sarcoma varied according to both histologic subtype and tumor size. Chemotherapy demonstrated directionally harmful associations in smaller undifferentiated pleomorphic sarcoma tumors and in intermediate- and large-sized fibromyxosarcoma, whereas dedifferentiated liposarcoma, epithelioid sarcoma, and synovial sarcoma showed no consistent survival benefit after adjustment. Larger (>10 cm) UPS tumors demonstrated a directionally protective association in matched analyses that attenuated after inverse probability weighting, suggesting that apparent benefit may reflect treatment selection or biologically favorable subsets rather than uniform chemotherapy responsiveness. Together, these findings support tumor size as a potential modifier of chemotherapy response rather than solely a prognostic feature, reinforcing the need for histology- and size-specific treatment considerations in localized disease. Clinically, these results suggest that decisions regarding chemotherapy in localized sarcoma may benefit from integrating tumor size with histologic subtype when counseling patients, rather than relying on size-based risk stratification alone. While some subgroup estimates were limited by size and should be interpreted cautiously, the overall pattern of size-dependent treatment associations across subtypes provides a relevant framework for individualized decision-making and highlights the need for prospective validation.

## Figures and Tables

**Table 1:** Baseline patients’ demographics, tumor characteristics, and treatment details by sarcoma subtype.

Characteristic	Undifferentiated Pleomorphic Sarcoma (*n* = 1627)	Dedifferentiated Liposarcoma (*n* = 615)	Fibromyxosarcoma (*n* = 1255)	Epithelioid Sarcoma (*n* = 156)	Synovial Sarcoma (*n* = 237)	*p*-Value
Age Group, n (%)						<0.001
Young (0–49 years)	10 (0.6%)	0 (0.0%)	48 (3.8%)	14 (9.0%)	36 (15.2%)	
Middle-aged (50–69 years)	74 (4.6%)	24 (3.9%)	137 (10.9%)	39 (25.0%)	85 (35.9%)	
Older (70–79 years)	578 (35.5%)	250 (40.7%)	500 (39.8%)	63 (40.4%)	85 (35.9%)	
Elderly (≥80 years)	965 (59.3%)	341 (55.5%)	570 (45.4%)	40 (25.6%)	31 (13.1%)	
Sex, *n* (%)						<0.001
Female	669 (41.1%)	193 (31.4%)	589 (46.9%)	66 (42.3%)	107 (45.1%)	
Male	958 (58.9%)	422 (68.6%)	666 (53.1%)	90 (57.7%)	130 (54.9%)	
Tumor Size, *n* (%)						<0.001
<5 cm	558 (34.3%)	85 (13.8%)	506 (40.3%)	65 (41.7%)	69 (29.1%)	
5–10 cm	631 (38.8%)	185 (30.1%)	508 (40.5%)	57 (36.5%)	101 (42.6%)	
>10 cm	438 (26.9%)	345 (56.1%)	241 (19.2%)	34 (21.8%)	67 (28.3%)	
Surgery, *n* (%)						<0.001
No	116 (7.1%)	32 (5.2%)	35 (2.8%)	25 (16.0%)	33 (13.9%)	
Yes	1511 (92.9%)	583 (94.8%)	1220 (97.2%)	131 (84.0%)	204 (86.1%)	
Radiotherapy, *n* (%)	624 (38.4%)	306 (49.8%)	583 (46.5%)	67 (42.9%)	89 (37.6%)	<0.001
No						
Yes	1003 (61.6%)	309 (50.2%)	672 (53.5%)	89 (57.1%)	148 (62.4%)	
Histologic Grade, *n* (%)						<0.001
Grade 1	28 (1.7%)	45 (7.3%)	194 (15.5%)	5 (3.2%)	6 (2.5%)	
Grade 2	191 (11.7%)	73 (11.9%)	473 (37.7%)	19 (12.2%)	49 (20.7%)	
Grade 3	505 (31.0%)	230 (37.4%)	212 (16.9%)	56 (35.9%)	117 (49.4%)	
Grade 4	903 (55.5%)	267 (43.4%)	376 (30.0%)	76 (48.7%)	65 (27.4%)	
Chemotherapy, *n* (%)						<0.001
No	1343 (82.5%)	521 (84.7%)	1153 (91.9%)	112 (71.8%)	124 (52.3%)	
Yes	284 (17.5%)	94 (15.3%)	102 (8.1%)	44 (28.2%)	113 (47.7%)	

**Table 2:** IPTW Sensitivity Analysis of Chemotherapy and Cause-Specific Survival by Histology and Tumor Size. Inverse probability of treatment weighting (IPTW) with stabilized weights was performed as a sensitivity analysis to account for measured treatment selection bias. Propensity scores were estimated within each histology and tumor size cohort using age group, sex, tumor grade (or high-grade classification when model instability occurred), surgery, and radiation therapy. Weighted Cox proportional hazards models with standard errors were used to estimate hazard ratios (HRs) for cancer-specific mortality. Tumor size categories were defined as <5 cm, 5–10 cm, and >10 cm.

Histology	Tumor Size	Cohort (N)	Events	Chemo (%)	HR (95% CI) of Cox Regression	*p*-Value of Cox Regression	HR (95% CI) After IPTW	*p*-Value After IPTW
Undifferentiated Pleomorphic Sarcoma	<5 cm	551	92	5.6	2.07 (1.00–4.28)	0.050	2.65 (1.19–5.92)	0.018
	5–10 cm	626	224	18.1	1.37 (1.00–1.87)	0.049	1.45 (1.03–2.04)	0.031
	>10 cm	424	213	32.8	0.72 (0.53–0.99)	0.041	0.82 (0.60–1.12)	0.211
Dedifferentiated Liposarcoma	<5 cm	69	18	8.7	2.17 (0.50–9.47)	0.303	1.90 (0.40–9.04)	0.418
	5–10 cm	185	74	15.1	1.24 (0.65–2.37)	0.508	0.78 (0.36–1.66)	0.514
	>10 cm	338	158	17.8	1.42 (0.97–2.08)	0.069	1.49 (0.96–2.29)	0.073
Fibromyxosarcoma	<5 cm	466	43	2.6	0.85 (0.12–6.16)	0.870	0.58 (0.08–4.02)	0.579
	5–10 cm	507	85	9.9	3.74 (2.30–6.10)	<0.001	4.47 (2.63–7.60)	<0.001
	>10 cm	212	75	18.9	1.72 (1.01–2.96)	0.048	2.16 (1.07–4.34)	0.031
Epithelioid Sarcoma	<5 cm	65	16	20.0	2.07 (0.65–6.60)	0.220	1.97 (0.58–6.70)	0.277
	5–10 cm	56	25	26.8	2.50 (1.00–6.24)	0.050	2.38 (0.92–6.18)	0.075
	>10 cm	29	22	51.7	1.30 (0.45–3.72)	0.631	0.89 (0.38–2.06)	0.782
Synovial Sarcoma	<5 cm	69	18	26.1	1.13 (0.40–3.18)	0.813	0.69 (0.21–2.27)	0.543
	5–10 cm	101	41	56.4	0.45 (0.19–1.05)	0.063	0.56 (0.22–1.44)	0.232
	>10 cm	60	47	55.0	1.20 (0.57–2.52)	0.631	1.38 (0.78–2.42)	0.268

## Data Availability

The datasets generated during and/or analysed during the current study are available from the corresponding author on reasonable request.

## Abbreviations

The following abbreviations are used in this manuscript:

UPS	Undifferentiated Pleomorphic Sarcoma
DDLPS	Dedifferentiated Liposarcoma
HR	Hazard Ratio
95% CI	95% Confidence Interval
